# Supplementary material for: Assessing the supply for a basic urban service demand-with a focus on water-energy management in Addis Ababa city
Source: PLoS One. 2021 Sep 7;16(9):e0249643. doi: 10.1371/journal.pone.0249643 (PMC8423246; doi:10.1371/journal.pone.0249643)
Supplement: S1 Table — (DOCX) [file pone.0249643.s001.docx]

S1 Table. Socio-economic data for Addis Ababa city (2005-2016)

| Socio-economic | Year | | | | | | | | | | | |
| --- | --- | --- | --- | --- | --- | --- | --- | --- | --- | --- | --- | --- |
|  | 2005 | 2006 | 2007 | 2008 | 2009 | 2010 | 2011 | 2012 | 2013 | 2014 | 2015 | 2016 |
| Population (million) | 2.68 | 2.73 | 2.79 | 2.85 | 2.91 | 2.98 | 3.05 | 3.12 | 3.19 | 3.27 | 3.35 | 3.43 |
| GDP (Billion ETB) | 0.02 | 0.03 | 0.03 | 0.03 | 0.04 | 0.04 | 0.05 | 0.06 | 0.06 | 0.07 | 0.09 | 0.10 |
| PCI (1000 ETB/capita) | 0.01 | 0.01 | 0.01 | 0.01 | 0.01 | 0.01 | 0.02 | 0.02 | 0.02 | 0.02 | 0.03 | 0.03 |
