# Supplementary material for: Assessing the supply for a basic urban service demand-with a focus on water-energy management in Addis Ababa city
Source: PLoS One. 2021 Sep 7;16(9):e0249643. doi: 10.1371/journal.pone.0249643 (PMC8423246; doi:10.1371/journal.pone.0249643)
Supplement: S2 Table — (DOCX) [file pone.0249643.s002.docx]

S2 Table. Water consumption in million cubic meters (MCM)

| Sector | Year | | | | |
| --- | --- | --- | --- | --- | --- |
|  | 2016 | 2017 | 2018 | 2019 | 2020 |
| Residential | 110 | 110 | 120 | 130 | 140 |
| Commercial | 30 | 30 | 30 | 30 | 40 |
| Industrial | 30 | 30 | 30 | 30 | 30 |
| Total | 170 | 170 | 180 | 190 | 210 |
