# Supplementary material for: Assessing the supply for a basic urban service demand-with a focus on water-energy management in Addis Ababa city
Source: PLoS One. 2021 Sep 7;16(9):e0249643. doi: 10.1371/journal.pone.0249643 (PMC8423246; doi:10.1371/journal.pone.0249643)
Supplement: S3 Table — (DOCX) [file pone.0249643.s003.docx]

S3 Table. Electric energy consumption in Giga watt hour (GWh) of Addis Ababa city sectors

| Sectors | Year | | | | |
| --- | --- | --- | --- | --- | --- |
|  | 2015 | 2016 | 2017 | 2018 | 2019 |
| Commercial | 561 | 708 | 903 | 989 | 1178 |
| Industrial | 1139 | 1169 | 1533 | 1683 | 1908 |
| Residential | 897 | 1022 | 1383 | 1414 | 1680 |
| Street-lighting | 8 | 14 | 14 | 17 | 18 |
