# Supplementary material for: Assessing the supply for a basic urban service demand-with a focus on water-energy management in Addis Ababa city
Source: PLoS One. 2021 Sep 7;16(9):e0249643. doi: 10.1371/journal.pone.0249643 (PMC8423246; doi:10.1371/journal.pone.0249643)
Supplement: S1 Appendix — (DOCX) [file pone.0249643.s004.docx]

**S1 Appendix**

**Table A1**. Water-energy consumption equation based on multivariate linear function (MLF)

| Water-energy sector | Function type | Equation |
| --- | --- | --- |
| Transport electric energy | MLF | -0.4*X_1_+49.8*X_2_+0.03 |
| Commercial electric energy | MLF | 1.5*X_1_+164.3*X_2_ |
| Residential electric energy | MLF | 1.9*X_1_+212.3*X_2_-8.9 |
| Industrial electric energy | MLF | 2*X_1_+210.4*X_2_-8.4 |
| Commercial water | MLF | -0.47*X_1_+0.001*X_2_-0.0012 |
| Industrial water | MLF | -0.07*X_1_+0.001*X_3_+0.007 |
| Residential water | MLF | -0.37*X_1_+0.004*X_3_-0.0003 |
